# Distinct cytokine profiles in malaria coinfections: A systematic review

**DOI:** 10.1371/journal.pntd.0011061

**Published:** 2023-01-30

**Authors:** Manas Kotepui, Wanida Mala, Pattamaporn Kwankaew, Kwuntida Uthaisar Kotepui, Frederick Ramirez Masangkay, Polrat Wilairatana

**Affiliations:** 1 Medical Technology Program, School of Allied Health Sciences, Walailak University, Tha Sala, Nakhon Si Thammarat, Thailand; 2 Department of Medical Technology, University of Santo Tomas, Manila, Philippines; 3 Department of Clinical Tropical Medicine, Faculty of Tropical Medicine, Mahidol University, Bangkok, Thailand; George Washington University School of Medicine and Health Sciences, UNITED STATES

## Abstract

**Background:**

Few data exist on the distinct cytokine profiles of individuals with malaria coinfections and other diseases. This study focuses on data collation of distinct cytokine profiles between individuals with malaria coinfections and monoinfections to provide evidence for further diagnostic or prognostic studies.

**Methods:**

We searched five medical databases, including Embase, MEDLINE, PubMed, Ovid, and Scopus, for articles on cytokines in malaria coinfections published from January 1, 1983 to May 3, 2022, after which the distinct cytokine patterns between malaria coinfection and monoinfection were illustrated in heat maps.

**Results:**

Preliminary searches identified 2127 articles, of which 34 were included in the systematic review. Distinct cytokine profiles in malaria coinfections with bacteremia; HIV; HBV; dengue; filariasis; intestinal parasites; and schistosomiasis were tumor necrosis factor (TNF), interferon (IFN)-γ, IFN-α, interleukin (IL)-1, IL-1 receptor antagonist (Ra), IL-4, IL-7, IL-12, IL-15, IL-17; TNF, IL-1Ra, IL-4, IL-10, IL-12, IL-18, CCL3, CCL5, CXCL8, CXCL9, CXCL11, granulocyte colony-stimulating factor (G-CSF); TNF, IFN-γ, IL-4, IL-6, IL-10, IL-12, CCL2; IFN-γ, IL-1, IL-4, IL-6, IL-10, IL-12, IL-13, IL-17, CCL2, CCL3, CCL4, G-CSF; IL-1Ra, IL-10, CXCL5, CXCL8, CXCL10; TNF, IL-2, IL-4, IL-6, IL-10; and TNF, IFN-γ, IL-4, IL-5, IL-10, transforming growth factor-β, CXCL8, respectively.

**Conclusion:**

This systematic review provides information on distinct cytokine profiles of malaria coinfections and malaria monoinfections. Further studies should investigate whether specific cytokines for each coinfection type could serve as essential diagnostic or prognostic biomarkers for malaria coinfections.

## Author summary

*Plasmodium falciparum*, *Plasmodium vivax*, *Plasmodium malariae*, *Plasmodium ovale*, and *Plasmodium knowlesi* infections all cause malaria, an acute febrile illness that is transmitted to people by the bites of infected female *Anopheles* mosquitoes. Following malaria infections in people, an individual’s immune responses are produced against the infection, leading to various clinical outcomes. The immune responses included groups of cytokines, small proteins that are secreted by immune system cells to help the body’s immune responses against infections. In malaria-endemic areas, malaria can coinfect with other several diseases as they have overlapping geographical distributions. Malaria coinfections with other diseases may lead to distinct cytokine production in infected patients. We, therefore, aimed to synthesize distinct cytokine profiles of individuals with malaria coinfections using a systematic review of the literature to provide insights into the current status of cytokine studies and identify research gaps. Based on the results of 34 studies, we synthesized that there were distinct cytokine profiles in malaria coinfections with bacteremia, intestinal parasites, and viruses. This information would provide the baseline data for further studies to investigate specific cytokines for each coinfection type that could serve as essential diagnostic or prognostic biomarkers for malaria coinfections.

## Introduction

*Plasmodium falciparum*, *Plasmodium vivax*, *Plasmodium malariae*, *Plasmodium ovale*, and *Plasmodium knowlesi* infections all cause malaria [1]. Currently, *P*. *falciparum* and *P*. *vivax* are the leading causes of malaria in the tropical and subtropical regions of many countries, with an estimated 247 million cases and 619 thousand deaths worldwide in 2021, according to the WHO’s most recent World malaria report [2]. While individuals with malaria may experience various clinical outcomes, such as severe, uncomplicated, or asymptomatic signs or symptoms [3], the host’s unique immunological response to the infection, which contributes to malaria pathophysiology, may account for these varying malaria signs and symptoms [4].

Immune responses in individuals with malaria are generally characterized by two primary subgroups of CD4 + T helper cells, including T helper 1 and 2 (Th1 and Th2). The disease is driven by a Th1 response early in the infection, with a gradual transition to a Th2 response as the disease develops [5,6]. Th1 cells release inflammatory cytokines such as interleukin-2 (IL-2), IL-6, IL-8, IL-12, IL-18, IL-23, interferon-gamma (IFN-γ), and tumor necrosis factor-alpha (TNF-α), which activate macrophages and cell-mediated immune responses [7]. Anti-inflammatory cytokines, including IL-4, IL-5, IL-10, and IL-13, are produced by Th2 cells, which in turn increase humoral immunity [7]. The second cytokine subgroup, chemoattractant cytokines, also known as chemokines, are essential in leukocyte migration, angiogenesis, and hematopoiesis [8]. During parasite infections, chemokine-guided migration permits leucocytes and immune cells to migrate to sites of infection or inflammation [9–12]. Chemokines are grouped into four subfamilies based on the locations of conserved cysteine residues near the N-terminus: CC, CXC, CX3C, and C [13]. The last cytokine subgroup, the colony-stimulating factors (CSFs), is a small family of homodimeric cytokines secreted by various cell types, such as macrophages, stromal cells, endothelial cells, and T lymphocytes, in the marrow microenvironment [14,15]. CSFs contribute significantly to blood formation, leukocyte function, and overall immunological competence. Granulocyte-CSF (G-CSF), macrophage-CSF (M-CSF), granulocyte/macrophage-CSF (GM-CSF), and IL-3 are all members of the CSF family. M-CSF and G-CSF are lineage-specific cytokines that affect macrophage and neutrophil survival, growth, differentiation, and function, respectively [16].

In malaria-endemic areas, malaria can coinfect with other tropical diseases as they have overlapping geographical distributions. Notably, several malaria coinfections with other tropical diseases have been documented, including dengue [17], hookworm [18], human African trypanosomiasis [19], typhoidal/nontyphoidal *Salmonella* [20], scrub typhus [21], visceral leishmaniasis [22], leptospirosis [23], Chikungunya [24], and the most recent coronavirus disease (COVID-19) or severe acute respiratory syndrome-coronavirus-2 (SARS-CoV-2) [25]. Because malaria coinfections may lead to severe diseases, such as malaria–dengue [26], malaria–human African trypanosomiasis [19], or malaria–nontyphoidal *Salmonella* coinfections [20], studies on malaria coinfections are crucial for understanding the role of other diseases in malaria pathophysiology and clinical outcomes. Although distinct cytokine profiles of malaria infection have been intensively examined over the last decade, studies on distinct cytokine profiles of individuals with malaria coinfections with other diseases remain underexplored. Therefore, this study focuses on data collation of distinct cytokine profiles of individuals with malaria coinfections to provide insights into the current status of cytokine studies and identify research gaps regarding whether cytokines can serve as disease markers or be used to track the progression of concurrent infections.

## Methods

### Search strategy and selection criteria

This study was registered with PROSPERO, CRDCRD42022331608. For this systematic review and meta-analysis, we adhered to the protocol outlined in the updated PRISMA 2020 guideline (S1 PRISMA Abstract Checklist; S1 PRISMA 2020 Checklist) [27]. We searched five medical databases (Embase, MEDLINE, PubMed, Ovid, and Scopus) for articles on cytokines in malaria coinfections published from January 1, 1983, to May 3, 2022. We also searched Google Scholar and the reference lists of the included studies and reviews for relevant studies. The search terms and complete eligibility requirements are detailed in the S1 Table. The search was restricted to articles written in English. Medical subject heading terms were used in the PubMed search, and their synonyms were modified for each database. For study selection, original papers reporting cytokines in malaria coinfections versus malaria monoinfections would be examined for eligibility. Meanwhile, animal studies, *in vitro* studies, reviews, conference abstracts, comments, and letters were excluded. We used EndNote version 20 software (Clarivate Analytics, Philadelphia, PA, USA) to remove duplicate articles and manually check the remaining for duplicates. The abstract screening and full-text selection were conducted independently by two authors (MK and KUK) following predetermined eligibility criteria (S1 Table). If the study selection was not unanimously agreed upon, a third author (WM) resolved this disagreement by making a final selection decision. For inclusion in meta-analysis, studies must report the mean and standard deviation (or median and interquartile range) of cytokines in malaria coinfections and monoinfections. In addition, the cytokines included in the meta-analysis must be reported in two or more studies, which is the requirement for a meta-analysis [28].

### Data extraction and quality assessment

Data extraction was performed using a standardized data extraction form. For cytokine levels reported in at least two studies, we extracted the mean (also standard deviation) or median (also interquartile range) from studies reporting cytokines in coinfections and malaria monoinfections. Only the study with the largest sample size was included if two or more articles reported the same setting and participants. The JBI’s critical appraisal tools were utilized to evaluate the studies’ quality and risk of bias. Two independent reviewers (KUK and WM) evaluated the quality of the included studies. Disagreements were resolved through discussion. Using Microsoft Excel 365, data extraction and study quality assessment were performed.

### Data syntheses

For qualitative synthesis, we illustrated the distinct cytokine patterns between malaria coinfection and malaria monoinfection in heat maps. Red, green, and yellow colors mean increased, comparable, and decreased levels of cytokines between the two groups, respectively. The distinct cytokine patterns between the two groups were then described in narrative form. For quantitative synthesis, we performed meta-analysis to combine the effect sizes of the mean difference (MD) of cytokine levels between malaria coinfections and malaria monoinfections using Stata version 17.0 (Stata Corporation, College Station, TX). The pooled estimates were calculated using random-effect models because all populations were assumed to be distinct. The DerSimonian–Laird method was used to estimate the variance between studies. In addition, subgroup analyses of coinfection types (bacteremia, viruses, and other parasites) were performed to identify cytokine differences unique to those populations. If mean and standard deviation were not reported by studies, the mean and standard deviation were calculated from the median and interquartile range as suggested elsewhere [29]. If studies reported the mean and standard deviation in several groups, we also combined means and standard deviation into one group, as described previously [30]. Using the Cochran Q statistic and I^2^ statistic, heterogeneity between studies was measured. Studies were deemed heterogeneous when the *P* value for the Cochran Q statistic was less than 0.05; the heterogeneity levels were classified as low (I^2^ < 25 percent), low to moderate (25% to 50%), moderate to high (50% to 75%), or high (>75%) [31]. To determine if the pooled estimates were robust, sensitivity analysis (leave-one-out method) was performed in which studies with extreme effect sizes and heterogeneity were excluded [32]. Using Egger’s test, the small-study effect was evaluated to detect publication bias. When the p value of Egger’s test was lower than 0.05, publication bias was suspected.

## Results

**Fig 1:**
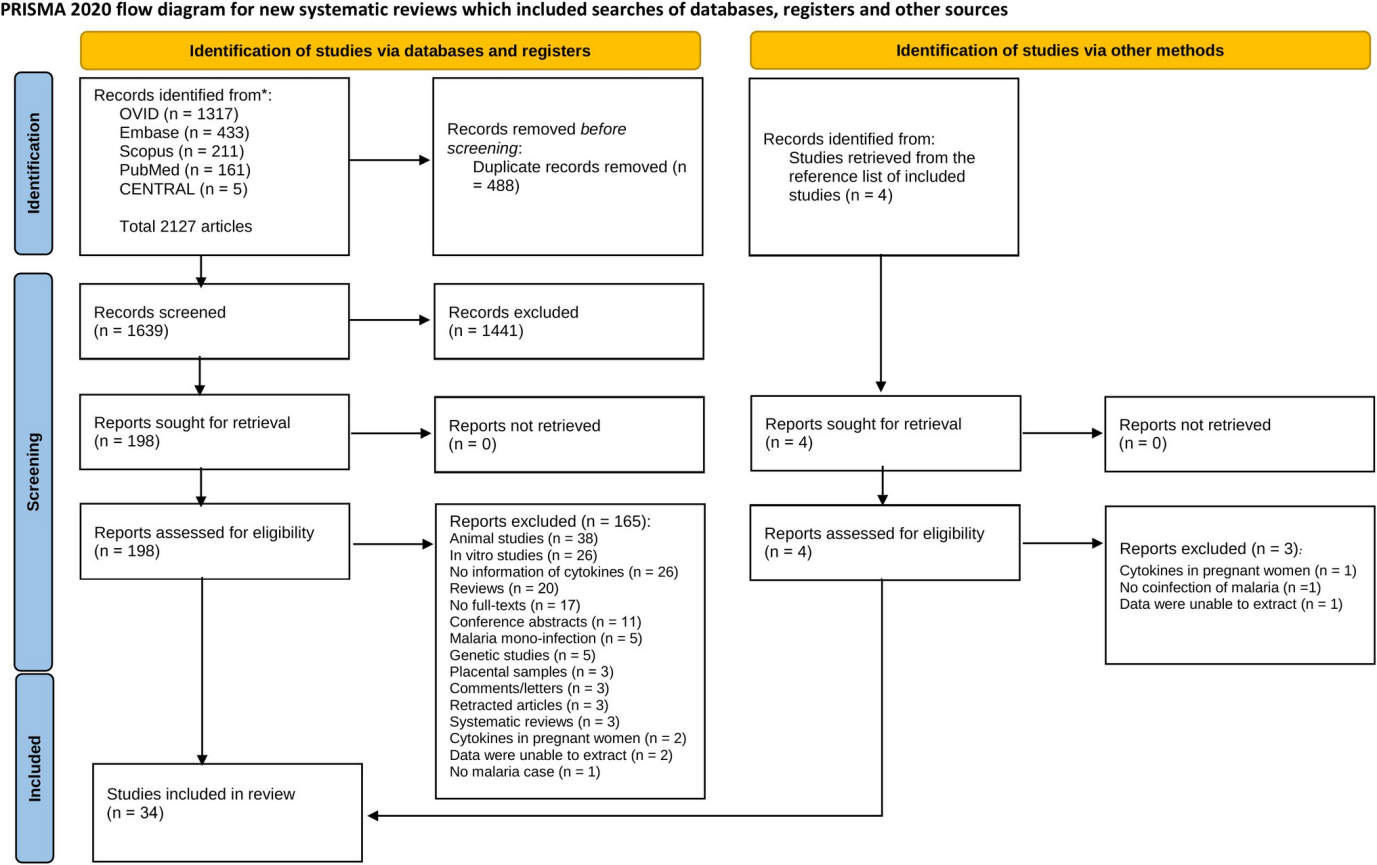
Flow diagram of the study selection process.

Our initial searches identified 2127 articles, of which 34 were included in the systematic review (Fig 1).

Notably, 82% (28 of 34) of the included studies were conducted in Africa and published between 2004 and 2022; 47% (16 of 34) were conducted using a cross-sectional study design; 23.5% (8 of 34) were prospective observational studies; and the remaining 29.4% (10 of 34) were cohort, case-control, retrospective observational, and clinical trial studies. Approximately 79.4% (27 of 34) of the studies enrolled patients infected with *P*. *falciparum*, and the remaining studies enrolled patients infected with other species. While 52.9% (18 of 34) of the studies reported malaria coinfections with other parasites, 38.2% (13 of 34) reported malaria coinfections with viruses. Table 1 provides a summary of the included studies, whereas S2 Table presents the details of the studies included in the systematic review and meta-analysis. S3 Table presents the clinical characteristics, coinfection types, and age groups of each study. All 34 studies were assessed for their quality (S4 Table) and were included in the systematic review.

**Table 1:** Characteristics of the 34 studies included in the study.

Characteristics	N. (34 studies)	%
**Study designs**		
Cross-sectional studies	16	47.0
Prospective observational studies	8	23.5
Cohort studies	4	11.8
Case-control studies	3	8.82
Retrospective observational studies	2	5.88
Clinical trials	1	2.94
**Study areas**		
Africa	28	82.4
South America	6	17.6
***Plasmodium* spp.**		
*P*. *falciparum*	27	79.4
*P*. *vivax*	5	14.7
*P*. *falciparum/P*. *vivax*	1	2.94
*P*. *falciparum* (2 cases were mixed infections with *P*. *vivax* and with *P*. *malariae*)	1	2.94
**Participants**		
Children and adults	16	47.0
Children	9	26.5
Adults	9	26.5
**Clinical malaria**		
Both severe and uncomplicated malaria	13	38.2
Uncomplicated malaria	8	23.5
Asymptomatic malaria	7	20.6
Both uncomplicated and asymptomatic malaria	3	8.82
Severe malaria (cerebral malaria)	1	2.94
Not specified	2	5.88
**Malaria coinfections**		
Parasites	18	52.9
Virus	13	38.2
Bacteria	1	2.86
Parasites and virus	1	2.94
Bacteria and virus	1	2.94
**Blood collections**		
Upon admission	21	61.8
Community survey	12	35.3
Community survey and upon admission	1	2.94
**Methods for cytokines**		
Bead-based assay	17	50.0
ELISA	17	50.0
**Blood sample for cytokines quantification**		
Plasma	26	76.5
Serum	7	20.6
Both plasma and serum	1	2.94

**Abbreviation:** ELISA, Enzyme-linked immunosorbent assay

**Fig 2:**
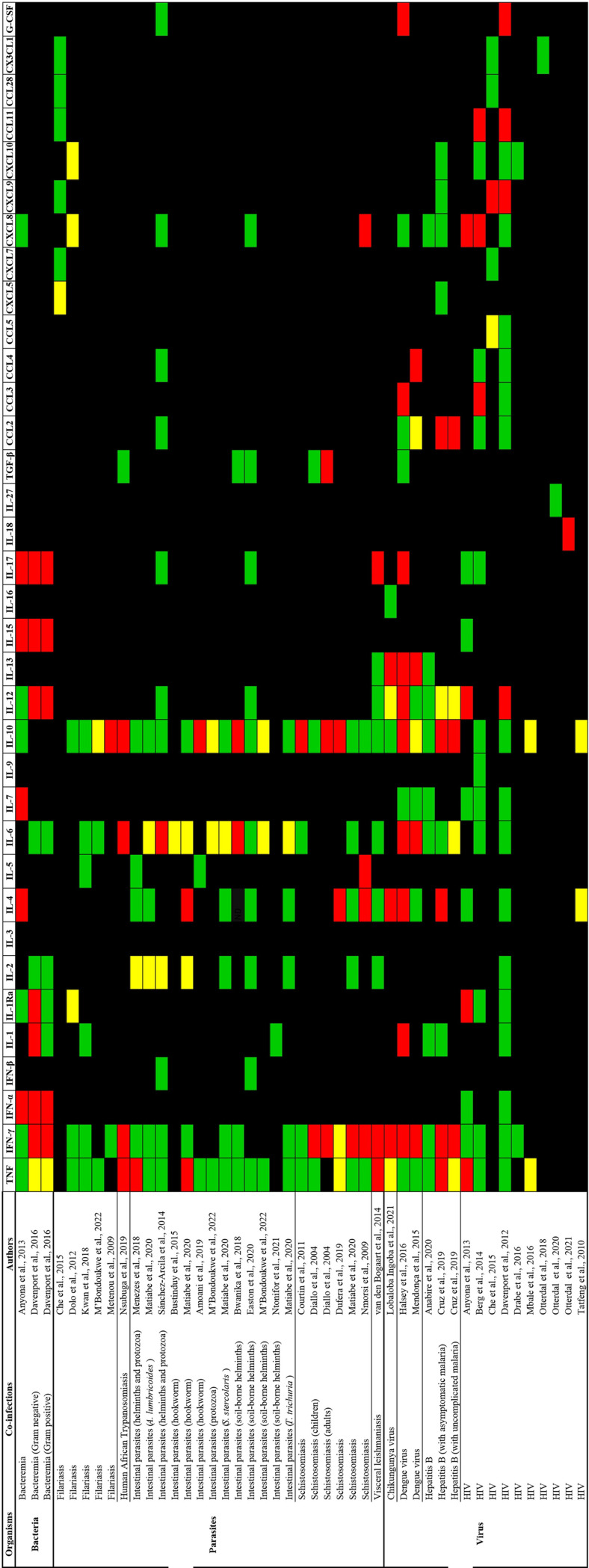
Distinct cytokine profiles in malaria coinfections versus malaria monoinfections. Red, green, and yellow indicate increased, comparable, and decreased cytokine levels between the two groups, respectively. References in the figure [33–57,59–61,63–65,83].

Among the 34 studies included in the systematic review, we identified 35 cytokines tested for their distinct expression in malaria coinfections compared to malaria monoinfections. These cytokines included TNF, IFN-γ, IFN-α, IFN-β, IL-1, IL-1Ra, IL-2, IL-3, IL-4, IL-5, IL-6, IL-7, IL-9, IL-10, IL-12, IL-13, IL-15, IL-16, IL-17, IL-18, IL-27, TGF-β, CCL2, CCL3, CCL4, CCL5, CXCL5, CXCL7, CXCL8, CXCL9, CXCL10, CCL11, CCL28, CX3CL1, and G-CSF. For malaria coinfections, we grouped cytokines into three domains: bacteria (Gram-positive and negative), parasites (filariasis, human African trypanosomiasis, intestinal parasites, schistosomiasis, and visceral leishmaniasis), and viruses (Chikungunya virus, dengue virus, hepatitis B, and HIV). Fig 2 shows distinct cytokine profiles in malaria coinfections versus malaria monoinfections.

### Malaria coinfections with bacteria

Two studies [33,34] reported distinct cytokine profiles in malaria coinfections with bacteremia. Distinct cytokine profiles were TNF, IFN-γ, IFN-α, IL-1, IL-1, Ra, IL-4, IL-7, IL-12, IL-15, and IL-17. Significantly increased levels of IFN-α, IL-15, and IL-17 were reported in coinfections compared to malaria monoinfection, whether Gram-positive or Gram-negative bacteremia coinfections. While IL-1 and IL-1Ra levels were significantly increased only in Gram-negative bacteremia coinfections, IFN-γ and IL-12 levels were significantly increased in both Gram-positive or Gram-negative bacteremia coinfections, as reported by Davenport et al. [34], but Anyona et al. [33] reported comparable levels of these cytokines between the two groups. Only Anyona et al. [33] investigated the significant increase in IL-4 and IL-7 levels in malaria and bacteremia coinfections compared to malaria monoinfections. TNF levels were decreased in coinfections, as reported by Davenport et al. [34], but Anyona et al. [33] reported comparable levels of this cytokine between the two groups.

### Malaria coinfections with viruses

Distinct cytokine profiles in malaria and HIV coinfections were TNF, IL-1Ra, IL-4, IL-10, IL-12, IL-18, CCL3, CCL5, CXCL8, CXCL9, CXCL11, and G-CSF; IL-12 [33,35], CXCL9 [35,36], CXCL11 [35,37], IL-18 [38], and G-CSF [35] levels were significantly increased in coinfections compared to malaria monoinfections. IL-1Ra was significantly increased in coinfection compared to malaria monoinfection, as reported by Anyona et al. [33], but comparable levels of these cytokines were reported by other studies [35,37]. CCL3 and CXCL8 were significantly increased in coinfections compared to malaria monoinfection, as reported by Berg et al. [37], but comparable levels of these cytokines were reported by Davenport et al. [35]. A significant decrease in IL-10 levels in malaria and HIV coinfections compared to malaria monoinfections was observed in two studies [39,40]. TNF [39] and IL-4 [40] levels were significantly decreased in malaria and HIV coinfections compared to malaria monoinfections.

Distinct cytokine profiles in malaria and HBV coinfections were TNF, IFN-γ, IL-4, IL-6, IL-10, IL-12, and CCL2. IFN-γ, IL-10, and CCL2 levels were significantly increased in coinfections compared to malaria monoinfection, as observed by Cruz et al. [41]. TNF levels were significantly increased in asymptomatic malaria and HBV coinfections, but their levels were significantly decreased in uncomplicated malaria and HBV coinfections [41]. IL-10 levels were increased, as observed by Cruz et al. [41], but comparable levels were demonstrated by Anabire et al. [42]. The IL-4 level was increased in asymptomatic malaria and HBV coinfections [41]. While a significant decrease in TNF levels was found in uncomplicated malaria and HBV coinfections [41], TNF levels were increased in asymptomatic malaria and HBV coinfections compared to malaria monoinfections. Anabire et al. [42] also reported comparable TNF levels in asymptomatic malaria compared to malaria monoinfections [42].

Distinct cytokine profiles in malaria and dengue coinfections were IFN-γ, IL-1, IL-4, IL-6, IL-10, IL-12, IL-13, IL-17, CCL2, CCL3, CCL4, and G-CSF. IFN-γ, IL-6, and IL-13 levels were significantly increased in coinfections compared to malaria monoinfections, as reported by two studies [43,44]. IL-4, IL-10, and IL-12 levels were significantly increased in coinfections compared to malaria monoinfections, as reported by Halsey et al. [43], but comparable levels of these cytokines were reported by Mendonça et al. [44]. IL-1, IL-a7, CCL3, and G-CSF levels were significantly increased in coinfections compared to malaria monoinfections, as reported by Halsey et al. [43], but CCL4 levels were significantly increased in coinfections compared to malaria monoinfections, as reported by Mendonça et al. [44]. Only IL-10 and CCL2 levels were significantly decreased in coinfections compared to malaria monoinfections, as reported by Mendonça et al. [44].

Distinct cytokine profiles in malaria and CHIKV coinfections were IFN-γ, IL-4, IL-12, and IL-13. IFN-γ, IL-4, and IL-13 levels were significantly increased in coinfections; meanwhile, TNF and IL-12 were significantly decreased in coinfections compared to malaria monoinfections as reported by Lobaloba Ingoba et al. [45].

### Malaria coinfections with other parasites

Distinct cytokine profiles in malaria and filariasis coinfections were IL-1Ra, IL-10, CXCL5, CXCL8, and CXCL10. Only IL-10 levels increased significantly in coinfections, according to Metenou et al. [46]; meanwhile, significantly decreased IL-1Ra, CXCL8, and CXCL10 levels in coinfections were reported by Dolo et al. [47]. In addition, significant decreases in IL-10 [48] and CXCL5 [36] levels were observed in coinfections compared to malaria monoinfections. No difference in TNF, IFN-γ, IL-1, IL-5, IL-6, and IL-10 levels between coinfections and malaria monoinfections as reported by Kwan et al. [49].

Distinct cytokine profiles in malaria and human African trypanosomiasis coinfections were TNF, IFN-γ, IL-6, and IL-10. TNF, IFN-γ, IL-6, and IL-10 levels increased significantly in coinfections compared to malaria monoinfections, according to Nsubuga et al. [50]. Distinct cytokine profiles in malaria and visceral leishmaniasis coinfections were TNF and IFN-γ. According to van den Bogaart et al. [51], the coinfection group had significantly higher TNF and IFN-γ levels than the malaria monoinfection group.

Distinct cytokine profiles in malaria and intestinal parasite coinfections were TNF, IL-2, IL-4, IL-6, and IL-10. While TNF levels were significantly increased in the coinfection group, IL-2 levels were significantly decreased compared to the malaria monoinfection group in two studies [52,53]. IL-4 levels were significantly increased only in hookworm coinfections in one study [52]. Although IL-6 levels were significantly increased in coinfections in two studies [54,55], its levels were significantly decreased in coinfections in another two studies [48,52]. Although IL-10 levels were significantly increased in coinfections in two studies [54,56], its levels were significantly decreased in coinfections, according to M’Bondoukwe et al. [48]. No difference in TNF, IFN-β, IL-2, IL-4, IL-6, IL-10, IL-12, IL-10, and TGF-β levels between coinfections and malaria monoinfections as reported by Easton et al. [57].

Distinct cytokine profiles in malaria and schistosomiasis coinfections were TNF, IFN-γ, IL-4, IL-5, IL-10, TGF-β, and CXCL8. TNF levels were significantly decreased in coinfections, according to Dufera et al. [58]. Three studies [52,59,60] reported that IFN-γ levels increased strikingly in coinfections, but this effect reversed, according to Dufera et al. [58]. The levels of IL-4, IL-5, and IL-10 were significantly increased in coinfections, according to two studies [58,60], Nmorsi et al. [60], and three studies [58,59,61], respectively. TGF-β [59] and CXCL8 [60] levels were also significantly higher in coinfections than in malaria monoinfections.

### Meta-analysis of cytokine profiles

The meta-analysis results revealed differences in cytokine profiles between malaria coinfections and monoinfections (Table 2). Compared to malaria monoinfection, IFN-γ and CXCL8 levels were significantly increased in malaria coinfections [(*P* < 0.001, MD: 26.10 pg/mL, 95% CI: −21.81–−10.67 pg/mL, I^2^: 99.21%, 5 studies) and (*P* < 0.001, MD: 366.02 pg/mL, 95% CI: 292.1–439.94 pg/mL, I^2^: 99.80%, 3 studies), respectively]. However, TNF levels were significantly decreased in malaria coinfections (*P* < 0.001, MD: −16.24 pg/mL, 95% CI: −21.81–−10.67 pg/mL), I^2^: 98.96%, 8 studies). Subgroup analysis of coinfection types revealed the following effects: IFN-γ, CXCL8, and IL-4 levels were increased in malaria and other parasite coinfections compared to malaria monoinfection (MD: 6.00 pg/mL, 95% CI: 5.54–6.46 pg/mL; MD: 1662 pg/mL, 95% CI: 1555.68–1768.32 pg/mL; MD: 10 pg/mL, 95% CI: 7.43–12.57 pg/mL, respectively), but IL-6 levels were decreased in malaria and other parasite coinfections (MD: −103.60 pg/mL, 95% CI: −193.71–(−13.49) pg/mL). Notably, no differences were observed in other cytokine levels—IL-10, CCL2, CCL3, IL-10 IL-1, IL-7 IL-12, and IL-17—between malaria coinfections and monoinfections (*P* > 0.05). The difference in cytokine levels between malaria and bacteria coinfections and malaria monoinfections could not be analyzed due to the lack of studies reporting the mean (and standard deviation) or median (and range) of cytokines.

**Table 2:** Meta-analysis results of cytokines between coinfections and malaria monoinfections.

Cytokines	*P* overall	MD (pg/mL)	95% CI (pg/mL)	I^2^	Egger’s test	Outlier (s)	No. of studies included in meta-analysis
**TNF**	**< 0.001**	**-16.24**	**-21.81-(-10.67)**	**98.96**	**< 0.001**	**Absence**	**8** [37,39,41–43]
Bacteria		ND	ND	ND	ND	ND	ND
Viruses		-8.03	-16.37–0.30	90.65			5 [37,39, 41–43]
Other parasites		-32	-41.37-(-22.64)	99.68			3 [48,56,60]
**IFN-γ**	**< 0.001**	**26.10**	**13.93–38.28**	**99.21**	**< 0.001**	**Presence**	**5 [37,41–43,60]**
Bacteria		ND	ND	ND	ND	ND	ND
Viruses		63.62	-6.11–133.5	99.35			4 [37,41–43]
Other parasites		6	5.54–6.46	ND			1 [60]
**IL-10**	**0.94**	**1.86**	**-48.47–52.19**	**99.63**	**< 0.001**	**Presence**	**9 [37,39–43,48,56,60]**
Bacteria		ND	ND	ND	ND	ND	ND
Viruses		-4.77	-172.92–163.38	99.73			6 [37,39–43]
Other parasites		109.56	-262.17–43.05	98.25			3 [48,56, 60]
**CXCL8**	**< 0.001**	**366.02**	**292.1–439.94**	**99.80**	**< 0.001**	**Absence**	**3** [37,43,60]
Bacteria		ND	ND	ND	ND	ND	ND
Viruses		7.83	-12.36–28.01	98.27			2 [37,43]
Other parasites		1662	1555.68–1768.32	ND			1 [60]
**CCL3**	**0.35**	**1.31**	**-1.45–4.08**	**98.35**	**< 0.001**	**ND**	**2 [37, 43]**
Bacteria		ND	ND	ND	ND		ND
Viruses		1.31	-1.45–4.08	98.35	< 0.001		2 [37,43]
Other parasites		ND	ND	ND	ND		ND
**CCL2**	**0.38**	**0.07**	**-0.09–0.22**	**94.77**	**< 0.001**	**Absence**	**3 [37,41,43]**
Bacteria		ND	ND	ND			ND
Viruses		0.07	-0.09–0.22	94.77			3 [37,41,43]
Other parasites		ND	ND	ND			ND
**IL-17**	**0.43**	**6.33**	**-9.52–22.17**	**95.49**	**0.107**	**ND**	**2 [37,43]**
Bacteria		ND	ND	ND			ND
Viruses		6.33	-9.52–22.17	95.49			2 [37,43]
Other parasites		ND	ND	ND			ND
**IL-12**	**0.16**	**-7.80**	**-18.76–3.17**	**98.47**	**0.001**	**Presence**	**3 [37,41,43]**
Bacteria		ND	ND	ND			ND
Viruses		-7.80	-18.76–3.17	98.47			3 [37,41,43]
Other parasites		ND	ND	ND			ND
**IL-7**	**0.39**	**2.27**	**-2.93–7.47**	**95.44**	**< 0.001**	**Absence**	**3 [37,42,43]**
Bacteria		ND	ND	ND			ND
Viruses		2.27	-2.93–7.47	95.44			3 [37,42,43]
Other parasites		ND	ND	ND			ND
**IL-1**	**0.71**	**-0.9**	**-5.64–3.83**	**85.37**	**0.241**	**Absence**	**3 [41,42,62]**
Bacteria		ND	ND	ND			ND
Viruses		-1.47	-6.17–3.24	91.69			2 [41,42]
Other parasites		17.93	-8.90–44.76	ND			1 [62]
**IL-4**	**0.09**	**11.06**	**-1.87–24.00**	**97.36**	**0.830**	**Presence**	**3 [41,43,60]**
Bacteria		ND	ND	ND			ND
Viruses		11.63	-14.56–37.81	98.68			2 [41,43]
Other parasites		10	7.43–12.57	ND			1 [60]
**IL-6**	**0.1**	**-23.68**	**-51.70–4.35**	**91.50**	**0.005**	**Presence**	**5 [37,41–43,48]**
Bacteria		ND	ND	ND			ND
Viruses		-17.51	-45.42–10.41	92.83			4 [37,41–43]
Other parasites		-103.60	-193.71-(-13.49)	ND			1 [48]

ND: Not determined due to no study for analysis or the number of studies for analysis were less than three.

### Cytokine levels in malaria coinfection compared to other disease monoinfections

Data on cytokine levels between malaria coinfection and other disease monoinfections were available in 17 studies [34,38,41–44,49,51,53–56,61–64]. Distinct cytokine profiles between malaria coinfection and other disease monoinfections are shown in S1 Fig. For malaria and parasite coinfections, Nsubuga et al. [50] showed increased TNF levels in malaria coinfections compared to human African trypanosomiasis monoinfection. For intestinal parasite infections, increased TNF [53,55], IL-1 [55,62], IL-6 [54,55], IL-10 [53–56], CCL2 [55] and decreased IL-4 [53], IL-17 [55], TGF-β [54] were observed in malaria coinfections compared to intestinal parasite monoinfection. IL-2 [53,55] and IL-5 [53,56] levels were observed as either lower or higher in malaria coinfections compared to intestinal parasite monoinfection. Courtin et al. [61] reported increased IL-10 levels in malaria coinfections compared to schistosomiasis monoinfection. van den Bogaart et al. [51] reported decreased IL-12, IL-13, and IL-17 levels in malaria coinfections compared to visceral leishmaniasis monoinfection.

For malaria and virus coinfections, increased TNF, IFN-γ, IL-6, and CCL4 levels [44] and decreased IL-4, IL-7 [43,44], IL-12 [44], TGF-β, and CCL3 levels [43] were observed in malaria coinfections compared to dengue monoinfection. Mendonça et al. [44] demonstrated increased TNF, IFN-γ, IL-6, and CCL4 levels and decreased IL-4, IL-7, and IL-12 levels in malaria coinfections compared to hepatitis B monoinfection. Increased IFN-γ, CXCL10, CX3CL1, IL-18, and IL-27 levels were observed in malaria coinfections compared to HIV monoinfection [38,63–65].

### Cytokine levels in malaria monoinfection compared to other disease monoinfection

Data on cytokine levels between malaria monoinfection and other disease monoinfections were available in 15 studies [40–45,50,53–56,62–65]. Distinct cytokine profiles between malaria monoinfection and other disease monoinfections are shown in S2 Fig. Nsubuga et al. [50] showed decreased IFN-γ, IL-6, and IL-10 levels in malaria monoinfection compared to HIV monoinfection and human African trypanosomiasis. Increased TNF [55], IFN-γ [53], IL-1 [55,62], IL-2 [53,55], IL-6 [55], IL-10 [53–55], TGF-β [54], CCL2 [55], CCL3 [55], and G-CSF [55] levels and decreased IL-5 [56], IL-12 [55], IL-17 [55], and CCL11 [56] levels were observed in malaria monoinfection compared to intestinal parasite monoinfection.

Lobaloba Ingoba et al. [45] showed increased TNF, IL-12, and IL-16 levels and decreased IFN-γ and IL-4 levels in malaria monoinfection compared to Chikungunya virus monoinfection. Increased TNF [44], IFN-γ [43], IL-6 [43], IL-10 [43,44], IL-17 [43], CCL2 [44], and CCL3 [43] levels and decreased IL-7 [44] levels were observed in malaria monoinfection compared to dengue virus monoinfection. Either increased or decreased levels of IL-4, IL-12, and IL-13 were observed in malaria monoinfection compared to dengue virus monoinfection [43,44]. Cruz et al. [41] reported increased IL-4 levels and decreased TNF, IFN-γ, IL-6, and CXCL9 levels in malaria monoinfection compared to hepatitis B virus monoinfection. Increased IFN-γ [65], IL-4 [40], IL-10 [40], IL-27 [63], CXCL10 [65], and CX3CL1 [64] levels were observed in malaria monoinfection compared to HIV virus monoinfection.

### Discussion

The present study showed distinct cytokine profiles in malaria coinfections compared to malaria monoinfections. Malaria coinfections with bacteremia were characterized by distinct cytokine profiles, including elevated IFN-γ, IFN-α, IL-4, IL-7, IL-12, IL-15, and IL-17 levels and decreased TNF levels, indicating a strong cytokine response. This strong cytokine response caused by malaria and bacteremia coinfections could result in worse clinical outcomes [66]. Bacteremia coinfections with malaria could enhance the clinical severity of anemia and sepsis by decreasing cyclooxygenase (COX)-2 and prostaglandin E2 (PGE2) expression [33], and these mediators were associated with TNF-α, IFN-γ, and IL-10 production [67,68] in coinfected individuals. Notably, proinflammatory cytokines were found to be highest in Gram-negative bacteremia coinfections, followed by Gram-positive bacteremia coinfections, and lowest in malaria monoinfections, suggesting that this cytokine profile promotes parasite clearance [34]. In bacteremia coinfections, one of the proinflammatory cytokines, such as TNF, was decreased. Through a feedback mechanism, the decreased TNF levels in coinfected patients may be due to the counterregulatory activities of IFN-induced increased nitric oxide (NO) that downregulate nitric oxide synthase (NOS)-inducing TNF-α [69]. Davenport et al. [34] reported decreased TNF levels in malaria and bacteremia coinfections, but another study [33] reported comparable levels of this cytokine between coinfections and malaria monoinfection, suggesting contradictory results between studies.

Distinct cytokine profiles in malaria and HIV coinfections compared to malaria monoinfections were increased IL-12, CXCL9, CXCL11, IL-18, and G-CSF levels and decreased TNF and IL-4 levels. Most of the distinct cytokine profiles in malaria and HIV coinfections were chemokines that act as chemoattractant for leukocytes playing roles in malaria pathogenesis. Although the exact mechanism of these chemokines in malaria pathogenesis is unknown, increased levels of chemokines such as CXCL9, CXCL10, and CXCL11 have been proposed as predictive markers for HIV disease progression [70]. G-CSF is another chemokine that contributes to the proliferation and differentiation of neutrophil granulocytes [71]. Increased G-CSF levels were associated with severe falciparum malaria, indicating a defense mechanism against malaria parasites [72]. IL-12 is a potent immunomodulatory cytokine that increases cell-mediated and humoral immune responses to malaria parasites by inducing isotype switching [73]. IL-4 is a potent immunomodulatory cytokine that correlates with the severity of malaria hyperparasitaemia but not the severity of the disease [74]. Although TNF had been proposed as a prognostic biomarker of severe malaria, as its levels were increased in patients with severe malaria [75], this study showed that TNF levels were significantly decreased in malaria and HIV coinfections. Evidence of lower TNF levels in these coinfections remains unclear. Notably, evaluating several TNF receptors or cytokine networks rather than TNF alone could prove to be more reliable markers of TNF activity [37].

Distinct cytokine profiles in malaria and HBV coinfections were TNF, IFN-γ, IL-4, IL-6, IL-10, IL-12, and CCL2. TNF, IFN-γ, IL-6, and IL-12 are proinflammatory cytokines associated with malaria infection and severe malaria [75–77]. Meanwhile, IL-4 and IL-10 act as anti-inflammatory cytokines by modulating IFN-γ [78]. Elevated IFN-γ:IL-10 ratio was associated with increased disease severity [79]). Although TNF, IL-6, and IL-12 are proinflammatory cytokines associated with severe malaria [75,76], their levels have been reported to be decreased in malaria and HBV coinfections [41,80]. The reason for the decrease in these proinflammatory cytokines in malaria and HBV coinfections remains unclear. Owing to the low parasite density among coinfections, malaria and HBV coinfections did not appear to significantly affect inflammatory responses in patients, according to a previous study [42]. Nevertheless, Cruz et al. [41] explored cytokines in both asymptomatic and uncomplicated malaria and revealed differences in TNF levels in malaria coinfections compared with malaria monoinfection—TNF levels were increased in asymptomatic but decreased in uncomplicated malaria [41]. These results indicated that coinfection by HBV drives the reduction of systemic inflammation caused by malaria parasites [80]. CCL2 is a chemokine that influences CD4 + T lymphocytes to produce IL-4 cytokine [81]. Therefore, increased CCL2 levels in coinfections may indicate a modulatory effect of IL-4 to downregulate key proinflammatory cytokines such as TNF-α [82].

Distinct cytokine profiles in malaria and dengue coinfections were IFN-γ, IL-1, IL-4, IL-6, IL-10, IL-12, IL-13, IL-17, CCL2, CCL3, CCL4, and G-CSF. Malaria and dengue coinfections showed a significant increase in proinflammatory cytokines such as IFN-γ, IL-1, IL-6, and IL-12; Th2 cytokines such as IL-4; anti-inflammatory cytokines such as IL-10 and IL-13; and Th17 cytokines such as IL-17. In addition, the levels of several chemokines, such as CCL3, CCL4, and G-CSF, also increased. Notably, both malaria and dengue have been linked to strong activation of proinflammatory cytokines and Th1 cytokines [43]. Therefore, increased levels of proinflammatory immune markers in the coinfection of malaria and dengue may cause the synergistic activation of immunological pathways of cytokines. Cases of malaria and dengue coinfection also exhibited the highest values of IFN-γ and IL-6. A study suggested that dengue viruses can affect the immune response of individuals with malaria and dengue coinfections as the number of parasitemia was lower in patients with coinfections [44]. These observations corroborated our previous findings that individuals with malaria and dengue coinfections were at a higher risk of severe diseases than those with monoinfection [26].

The prevalence of malaria and CHIKV coinfections among febrile patients has been shown to vary with diagnostic tests for CHIKV infection, and coinfection occurred by chance [24]. The present study showed that the levels of proinflammatory cytokines such as IFN-γ and IL-12; Th2 cytokines such as IL-4; and anti-inflammatory cytokines such as IL-13 were increased in coinfections, but the levels of some proinflammatory cytokines, such as TNF and IL-12, were decreased in coinfections. These results indicated that TNF and IL-12 activities were suppressed among individuals with coinfections. Malaria monoinfection exhibited high TNF and IL-12 responses. Therefore, malaria and CHIKV coinfections might suppress viral replication [45] or inhibit the severity of parasite infection.

Malaria and other parasite coinfections demonstrated varying cytokine responses. Most of the cytokines that were investigated in malaria and other parasite coinfections were TNF, IFN-γ, IL-2, IL-4, IL-6, and IL-10. TNF levels were increased in malaria coinfections with human African trypanosomiasis [49] and intestinal parasites [52,53]. Meanwhile, IFN-γ levels were increased among individuals with human African trypanosomiasis [50], schistosomiasis [52,59,60], and visceral leishmaniasis [51]. Other cytokines such as IL-4, IL-6, and IL-10 were also elevated in coinfections compared to malaria monoinfection. These results indicated that malaria and other parasite coinfections exhibited stronger proinflammatory cytokine responses than malaria monoinfections. However, a small number of studies have investigated these cytokines, and not all studies have evaluated similar cytokines in coinfections. IFN-γ levels were distinctly increased among malaria and schistosomiasis coinfections in three studies [52,59,60]. Although IFN- γ can induce the production of TNF-α and other molecules, most studies have demonstrated that malaria and schistosomiasis coinfections have comparable TNF levels compared to malaria monoinfection. Therefore, other factors, such as age, intensity of infection, and hormones, could directly affect the balance between proinflammatory and anti-inflammatory cytokines in these types of coinfections [59]. IL-6 levels were decreased in most studies that investigated malaria and intestinal parasite coinfections ([48,52,83]. IL-6 has been implicated as a candidate marker for severe malaria, as its levels were increased in severe malaria compared to uncomplicated malaria [77]. Therefore, decreased IL-6 levels in patients with malaria and intestinal parasite coinfections might be a protective factor for disease severity among individuals with coinfections. The next common cytokine that studies evaluated in both malaria and other parasite coinfections was IL-10. IL-10 levels were elevated in human African trypanosomiasis [50], hookworm and soil-transmitted helminths [54,56], and schistosomiasis coinfections [58,59,61]. Again, distinct IL-10 levels were frequently observed among schistosomiasis coinfections compared to malaria monoinfection. This result indicates that IL-10 plays a role in regulating excessive inflammatory responses by its potent anti-inflammatory cytokines and could provide protection against severe disease [84].

The meta-analysis results based on the limited number of studies showed that IFN-γ and CXCL8 levels were significantly increased in malaria coinfections compared to malaria monoinfection. Meanwhile, TNF levels were significantly decreased in malaria coinfections compared to malaria monoinfection. Some limitations exist regarding the number of studies included in the systematic review. First, because the number of studies for each malaria coinfection type was small, the conclusion of distinct cytokine profiles provided in malaria coinfections with human African trypanosomiasis, visceral leishmaniasis, and Chikungunya compared to malaria monoinfection could not be made. Second, cytokine responses in the different malaria species were not analyzed because most studies reported *P*. *falciparum* coinfections. Third, considering data heterogeneity and the small number of studies that reported cytokine levels in each coinfection type, the analysis or synthesis of cytokine levels between children and adults, or severe and uncomplicated malaria, could not be performed. Because only a few studies have reported the cytokine levels in each severe malaria complication that may exert the difference in cytokine profile between malaria coinfection and malaria monoinfection, the evidence could not be synthesized. In addition, because the data on cytokine levels in each severe malaria complication were limited, additional research is required to determine these cytokine levels in various severe malaria complications.

## Conclusion

The systematic review revealed distinct cytokine profiles in malaria coinfections compared to malaria monoinfections. Further research is required to determine whether specific cytokines for each coinfection type could serve as useful diagnostic or prognostic biomarkers for malaria coinfections. Future investigations of the following factors will be crucial: IFN-α, IL-15, and IL-17 levels for malaria and bacteremia coinfections; IL-12 and CXCL9 levels for malaria and HIV coinfections; TNF and IL-10 levels for malaria and HBV coinfections; IFN-γ, IL-6, and IL-13 levels for malaria and dengue coinfections; IL-6 and IL-10 levels for malaria and intestinal parasite coinfections; and IFN-γ and IL-10 levels for malaria and schistosomiasis coinfections.

## Supporting information

S1 PRISMA 2020 Checklist (DOCX)

S1 Fig Distinct cytokine profiles in malaria coinfections versus other disease monoinfections. Red, green, and yellow indicate increased, comparable, and decreased cytokine levels between the two groups, respectively. References in the figure [34,38,41–44,49,51,53–56,61–64]. (TIF)

S2 Fig Distinct cytokine profiles in malaria monoinfections versus other disease monoinfections. Red, green, and yellow indicate increased, comparable, and decreased cytokine levels between the two groups, respectively. References in the figure [40–45,50,53–56,62–65].
(TIF)

S1 Table Search terms. (DOCX)

S2 Table Details of the studies included in the systematic review and meta-analysis. (XLSX)

S3 Table Clinical characteristics, coinfection types, and age groups of each study. (DOCX)

S4 Table Quality of the included studies. (XLSX)

S1 PRISMA Abstract Checklist (DOCX)
