# Supplementary figures and images for: Distinct cytokine profiles in malaria coinfections: A systematic review
Source: PLoS Negl Trop Dis. 2023 Jan 30;17(1):e0011061. doi: 10.1371/journal.pntd.0011061 (PMC9886258; doi:10.1371/journal.pntd.0011061)

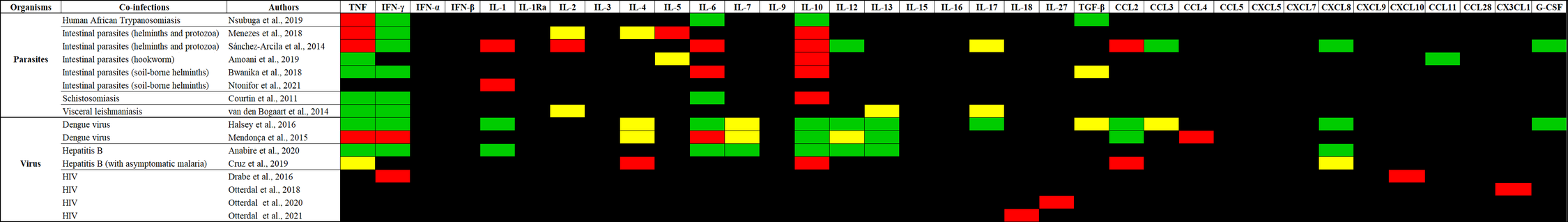

Supplement: S1 Fig — (TIF) [file pntd.0011061.s002.tif]

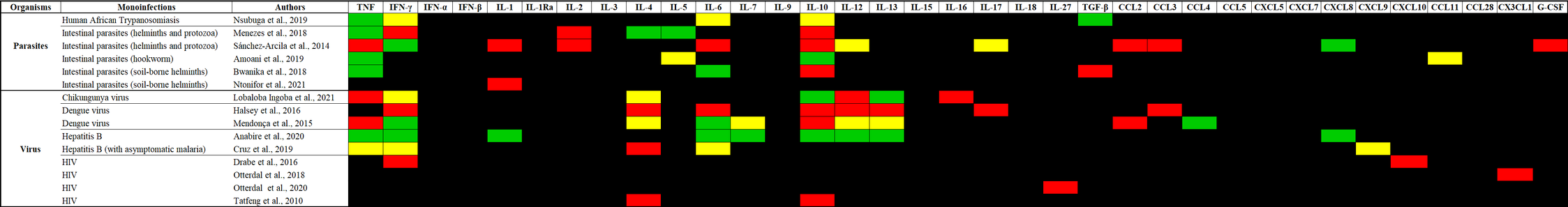

Supplement: S2 Fig — Red, green, and yellow indicate increased, comparable, and decreased cytokine levels between the two groups, respectively. References in the figure [40–45,50,53–56,62–65]. (TIF) [file pntd.0011061.s003.tif]
