# Supplementary material for: Distinct cytokine profiles in malaria coinfections: A systematic review
Source: PLoS Negl Trop Dis. 2023 Jan 30;17(1):e0011061. doi: 10.1371/journal.pntd.0011061 (PMC9886258; doi:10.1371/journal.pntd.0011061)
Supplement: S1 Table — (DOCX) [file pntd.0011061.s004.docx]

**Distinct cytokine profiles in malaria coinfections: A systematic review**

Manas Kotepui^1*^, Wanida Mala^1^, Pattamaporn Kwankaew^1^, Kwuntida Uthaisar Kotepui^1^, Frederick Ramirez Masangkay^2^, Polrat Wilairatana^3*^

^1^ Medical Technology, School of Allied Health Sciences, Walailak University, Tha Sala, Nakhon Si Thammarat, Thailand

^2^ Department of Medical Technology, University of Santo Tomas, Manila, Philippines;

^3^ Department of Clinical Tropical Medicine, Faculty of Tropical Medicine, Mahidol University, Bangkok, Thailand

**^*^Corresponding author**

Manas Kotepui: manas.ko@wu.ac.th, Tel.: +66954392469

Kwuntida Uthaisar Kotepui: [kwuntida.ut@wu.ac.th](mailto:kwuntida.ut@wu.ac.th)

Wanida Mala: wanida.ma@wu.ac.th

Pattamaporn Kwankaew: pattamaporn.kw@wu.ac.th

Frederick Ramirez Masangkay: frederick_masangkay2002@yahoo.com

Polrat Wilairatana; [polrat.wil@mahidol.ac.th](mailto:polrat.wil@mahidol.ac.th)

**Table S1. Search strategy**

**Embase**

**3 May 2022**

| No. | Query | Results |
| --- | --- | --- |
| #4 | #1 AND #2 AND #3 | 433 |
| #3 | 'co infect*' OR coinfect* OR concurrent | 257545 |
| #2 | 'cytokine'/exp OR cytokine OR 'chemokine'/exp OR chemokine | 1884406 |
| #1 | 'malaria'/exp OR malaria OR 'plasmodium'/exp OR plasmodium | 155366 |

**PubMed**

**3 May 2022**

| Search number | Query | Search Details | Results |
| --- | --- | --- | --- |
| 4 | #1 AND #2 AND #3 | ("malaria"[MeSH Terms] OR "malaria"[Title/Abstract]) AND ("coinfection"[MeSH Terms] OR "co-infection"[Title/Abstract] OR "coinfection"[MeSH Terms] OR "coinfection"[Title/Abstract] OR "co infect*"[Title/Abstract] OR "concurrent"[Title/Abstract]) AND ("cytokines"[MeSH Terms] OR "cytokine"[Title/Abstract] OR "chemokines"[MeSH Terms] OR "chemokine"[Title/Abstract]) | 161 |
| 3 | (((cytokine[MeSH Terms]) OR (cytokine[Title/Abstract])) OR (chemokine[MeSH Terms])) OR (chemokine[Title/Abstract]) | "cytokines"[MeSH Terms] OR "cytokine"[Title/Abstract] OR "chemokines"[MeSH Terms] OR "chemokine"[Title/Abstract] | 849,511 |
| 2 | (((((((co-infection[MeSH Terms]) OR (co-infection[Title/Abstract])) OR (coinfection[MeSH Terms])) OR (coinfection[Title/Abstract])) OR (co-infect*[MeSH Terms])) OR (co-infect*[Title/Abstract])) OR (concurrent[MeSH Terms])) OR (concurrent[Title/Abstract]) | "coinfection"[MeSH Terms] OR "co-infection"[Title/Abstract] OR "coinfection"[MeSH Terms] OR "coinfection"[Title/Abstract] OR "co infect*"[Title/Abstract] OR "concurrent"[Title/Abstract] | 167,001 |
| 1 | (malaria[MeSH Terms]) OR (malaria[Title/Abstract]) | "malaria"[MeSH Terms] OR "malaria"[Title/Abstract] | 100,602 |

**CENTRAL**

**3 May 2022**

**Step/Query/Search Details/Results**

#1 MeSH descriptor: [Malaria] explode all trees 3291

#2 (malaria):ti,ab,kw (Word variations have been searched) 7024

#3 ("Plasmodium"):ti,ab,kw (Word variations have been searched) 3092

#4 #1 OR #2 OR #3 7147

#5 MeSH descriptor: [Cytokines] explode all trees 21396

#6 MeSH descriptor: [Chemokines] explode all trees 1683

#7 MeSH descriptor: [Coinfection] explode all trees 218

#8 MeSH descriptor: [Coinfection] explode all trees 218

#9 (cytokine):ti,ab,kw (Word variations have been searched) 19471

#10 (chemokine):ti,ab,kw (Word variations have been searched) 2407

#11 (co-infect* OR coinfect* OR concurrent):ti,ab,kw (Word variations have been searched) 20450

#12 #5 OR #6 OR #9 OR #10 35843

#13 #7 OR #8 OR #11 20450

#14 #4 AND #12 AND #13 5

**Ovid**

**Step/Query/Search Details/Results**

1 malaria.m_titl. 9392

2 malaria.mp. [mp=title, abstract, full text, caption text] 53473

3 plasmodium.m_titl. 3500

4 plasmodium.mp. [mp=title, abstract, full text, caption text] 18422

5 cytokine.m_titl. 16263

6 cytokine.mp. [mp=title, abstract, full text, caption text] 346786

7 chemokine.m_titl. 5053

8 chemokine.mp. [mp=title, abstract, full text, caption text] 86509

9 "co-infect*".m_titl. 2046

10 co-infect*.mp. [mp=title, abstract, full text, caption text] 24898

11 "coinfect*".m_titl. 2563

12 coinfect*.mp. [mp=title, abstract, full text, caption text] 30490

13 concurrent.m_titl. 8235

14 concurrent.mp. [mp=title, abstract, full text, caption text] 318807

15 1 or 2 or 3 or 4 57853

16 5 or 6 or 7 or 8 379137

17 9 or 10 or 11 or 12 or 13 or 14 365357

18 15 and 16 and 17 1317

**Note:** Search All Ovid Journals (Abstracts only)

**Scopus**

3 May 2022

| **Databases** | **Search terms/Search strategy** | **Date** |
| --- | --- | --- |
| Scopus | ( TITLE-ABS-KEY ( malaria OR plasmodium ) ) AND ( TITLE-ABS-KEY ( cytokine OR chemokine ) ) AND ( TITLE-ABS-KEY ( co-infect* OR coinfect* OR concurrent ) )  Search results: 211 | 3 May 2022 |
