# Supplementary material for: Distinct cytokine profiles in malaria coinfections: A systematic review
Source: PLoS Negl Trop Dis. 2023 Jan 30;17(1):e0011061. doi: 10.1371/journal.pntd.0011061 (PMC9886258; doi:10.1371/journal.pntd.0011061)
Supplement: S3 Table — (DOCX) [file pntd.0011061.s006.docx]

**Distinct cytokine profiles in malaria coinfections: A systematic review**

Manas Kotepui^1*^, Wanida Mala^1^, Pattamaporn Kwankaew^1^, Kwuntida Uthaisar Kotepui^1^, Frederick Ramirez Masangkay^2^, Polrat Wilairatana^3*^

^1^ Medical Technology, School of Allied Health Sciences, Walailak University, Tha Sala, Nakhon Si Thammarat, Thailand

^2^ Department of Medical Technology, University of Santo Tomas, Manila, Philippines;

^3^ Department of Clinical Tropical Medicine, Faculty of Tropical Medicine, Mahidol University, Bangkok, Thailand

**^*^Corresponding author**

Manas Kotepui: manas.ko@wu.ac.th, Tel.: +66954392469

Kwuntida Uthaisar Kotepui: [kwuntida.ut@wu.ac.th](mailto:kwuntida.ut@wu.ac.th)

Wanida Mala: wanida.ma@wu.ac.th

Pattamaporn Kwankaew: pattamaporn.kw@wu.ac.th

Frederick Ramirez Masangkay: frederick_masangkay2002@yahoo.com

Polrat Wilairatana; [polrat.wil@mahidol.ac.th](mailto:polrat.wil@mahidol.ac.th)

**Table S3.** Clinical characteristics, types of co-infections, and age groups

| **Clinical malaria** | **Type of co-infections** | **age groups** |
| --- | --- | --- |
| NS (possibly asymptomatic) | Filariasis | Adults |
| Uncomplicated malaria | Filariasis | Children and adults |
| Asymptomatic malaria | Filariasis, soil-borne helminths, intestinal protozoa | Children and adults |
| Asymptomatic malaria | Intestinal parasites | NS |
| Asymptomatic malaria | Schistosomiasis | Children and adults |
| Asymptomatic malaria | Hookworm | Children and adults |
| Asymptomatic malaria | Hookworm | Children and adults |
| Asymptomatic malaria | Schistosomiasis | Children and adults |
| Both uncomplicated and asymptomatic malaria | Schistosomiasis | Children and adults |
| Both severe and uncomplicated malaria | Schistosomiasis | Children |
| Both uncomplicated and asymptomatic malaria | HIV and filariasis | Children and adults |
| Asymptomatic malaria | Hepatitis B | Adults |
| Both uncomplicated and asymptomatic malaria | Chronic hepatitis B | Adults |
| Asymptomatic malaria | Hepatitis B | Children and adults |
| Both severe and uncomplicated malaria | Bacteremia | Children |
| Both severe and uncomplicated malaria | Bacteria, HIV | Children |
| Both severe and uncomplicated malaria | Filariasis | Children |
| Uncomplicated malaria | Soil-borne helminths | Children |
| Uncomplicated malaria | Dengue virus | Adults |
| Both severe and uncomplicated malaria | HIV | Adults |
| Both severe and uncomplicated malaria | HIV | Children |
| Uncomplicated malaria | Human African Trypanosomiasis | Children and adults |
| Both severe and uncomplicated malaria | Intestinal helminths | Children and adults |
| Both severe and uncomplicated malaria | Soil-borne helminths | Children |
| Uncomplicated malaria | Chikungunya virus | Children and adults |
| Both severe and uncomplicated malaria | HIV | Adults |
| Both severe and uncomplicated malaria | HIV | Adults |
| Both severe and uncomplicated malaria | HIV | Adults |
| Severe malaria (cerebral malaria) | HIV | Children |
| Uncomplicated malaria | HIV | Adults |
| Both severe and uncomplicated malaria | Soil-borne helminths and schistosomiasis | Children |
| NS (asymptomatic or uncomplicated malaria) | Intestinal parasites | Children and adults |
| Both severe and uncomplicated malaria | Dengue virus | Children and adults |
| Uncomplicated malaria | HIV | NS |
| Uncomplicated malaria | Visceral leishmaniasis | Children and adults |
